# A Systematic Review with Meta-Analysis of the Effect of Resistance Training on Whole-Body Muscle Growth in Healthy Adult Males

**DOI:** 10.3390/ijerph17041285

**Published:** 2020-02-17

**Authors:** Pedro J. Benito, Rocío Cupeiro, Domingo J. Ramos-Campo, Pedro E. Alcaraz, Jacobo Á. Rubio-Arias

**Affiliations:** 1 LFE Research Group, Department of Health and Human Performance, Faculty of Physical Activity and Sport Science-INEF, Universidad Politécnica de Madrid, 28040 Madrid, Spain; rocio.cupeiro@upm.es (R.C.); ja.rubio@upm.es (J.Á.R.-A.); 2 Department of Physical Activity and Sports Sciences, Faculty of Sports, UCAM, Catholic University San Antonio, 30107 Murcia, Spain; djramos@ucam.edu (D.J.R.-C.); palcaraz@ucam.edu (P.E.A.); 3 UCAM Research Centre for High Performance Sport, Catholic University San Antonio, 30107 Murcia, Spain

**Keywords:** hypertrophy, fat-free mass, skeletal muscle mass, lean muscle mass, resistance training

## Abstract

We performed a systematic review and meta-analysis to study all published clinical trial interventions, determined the magnitude of whole-body hypertrophy in humans (healthy males) and observed the individual responsibility of each variable in muscle growth after resistance training (RT). Searches were conducted in PubMed, Web of Science and the Cochrane Library from database inception until 10 May 2018 for original articles assessing the effects of RT on muscle size after interventions of more than 2 weeks of duration. Specifically, we obtain the variables fat-free mass (FMM), lean muscle mass (LMM) and skeletal muscle mass (SMM). The effects on outcomes were expressed as mean differences (MD) and a random-effects meta-analysis and meta-regressions determined covariates (age, weight, height, durations in weeks…) to explore the moderate effect related to the participants and characteristics of training. One hundred and eleven studies (158 groups, 1927 participants) reported on the effects of RT for muscle mass. RT significantly increased muscle mass (FFM+LMM+SMM; Δ1.53 kg; 95% CI [1.30, 1.76], *p* < 0.001; I^2^ = 0%, *p* = 1.00). Considering the overall effects of the meta-regression, and taking into account the participants’ characteristics, none of the studied covariates explained any effect on changes in muscle mass. Regarding the training characteristics, the only significant variable that explained the variance of the hypertrophy was the sets per workout, showing a significant negative interaction (MD; estimate: 1.85, 95% CI [1.45, 2.25], *p* < 0.001; moderator: -0.03 95% CI [−0.05, −0.001] *p* = 0.04). In conclusion, RT has a significant effect on the improvement of hypertrophy (~1.5 kg). The excessive sets per workout affects negatively the muscle mass gain.

## 1. Introduction

Achieving a proper muscle mass is a key factor for sports performance as well as for attaining a good body image. Furthermore, muscle is increasingly being recognized as a key tissue for the maintenance of an adequate health status, not only regarding movement and posture but also as a regulator of inter-organ crosstalk for energy and protein metabolism throughout the body [1]. Therefore, improving lean body mass and preventing muscle loss should be crucial in promoting overall health and in achieving the targeted performance level [1,2].

In this context, being able to predict changes in body composition would be very useful for training and conditioning and for health professionals, both in the non-clinical and the clinical fields, helping them to set appropriate goals as well as to correctly assess the progression during interventions. However, despite its importance, several limitations arise regarding muscle hypertrophy, defined as an increase in the size of muscle tissue [3]. On the one hand, no consensus exists for quantifying this increase in muscle tissue, since different methods are employed to measure it (fat-free mass, lean muscle mass or skeletal muscle mass). On the other hand, another limitation for trainers and practitioners is that with current knowledge and research it is difficult to predict how much muscle mass can be gained.

Several studies have already designed estimation equations to predict weight evolution throughout a weight loss programme [4,5], and although an intrinsic uncertainty and error margin of those estimations are always present, the same method of evaluation for muscle gain is necessary as we have already pointed out. In this sense, two pioneer works have paved the way to the prediction of muscle hypertrophy. Firstly, the meta-analysis by Morton et al. [6] has shown how supplementation can influence muscle hypertrophy, explicitly reporting the weight of this variable (i.e., supplementation) over the levels of muscle gain. Secondly, based on the models proposed by Hall et al. [5], Torres et al. created a mathematical model to predict muscle hypertrophy in humans. Nevertheless, the work by Morton et al. [6] focuses on the effect of protein intake on muscle gain, without addressing the effect of training without protein supplementation; while the model created by Torres et al. is a theoretical model focused on fat loss and lean mass gain in obese people, not determining the effect of the different training parameters. In fact, the authors declare that further models may be developed and redefined to include parameters they had not addressed [7]. Therefore, it seems that a really integrative model, taking into account all the variables that influence muscle hypertrophy, is currently lacking.

In this respect, muscle hypertrophy in humans is influenced by numerous variables, from those inherent to exercise such as mechanical tension, metabolic stress, muscle damage, training volume and intensity [8,9]; to those being specific to the individual (age, sex, previous training status, etc.) [10], or those related to energy balance and protein metabolism [6,7]. Consequently, any study aiming to analyse and estimate muscle hypertrophy in humans must precisely address all confounding factors that may completely alter the final results.

As mentioned above, only two studies have attempted to estimate the changes in muscle mass after a training intervention [6,7]. However, none of them handle the effect of these confounding factors that we have mentioned. Therefore, the effect of an isolated training programme on muscle hypertrophy, the variation in its design, age or resistance training (RT) experience, are currently unknown. Thus, the aims of the present study were: (i) to quantify the impact of strength training on human whole-body muscle hypertrophy, and (ii) to analyse training factors which could moderate the human muscle growth in men.

## 2. Materials and Methods 

### 2.1. Study Design 

The methodological process was based on the recommendations indicated by the PRISMA declaration [11]. The eligibility criteria were established by the authors. For the meta-analysis, research that studied resistance training was considered. The study was preregistered in the International Prospective Register of Systematic Review (PROSPERO) with the following registered number: CRD42018106274. 

### 2.2. Data Sources and Search Profile 

**Figure 1:**
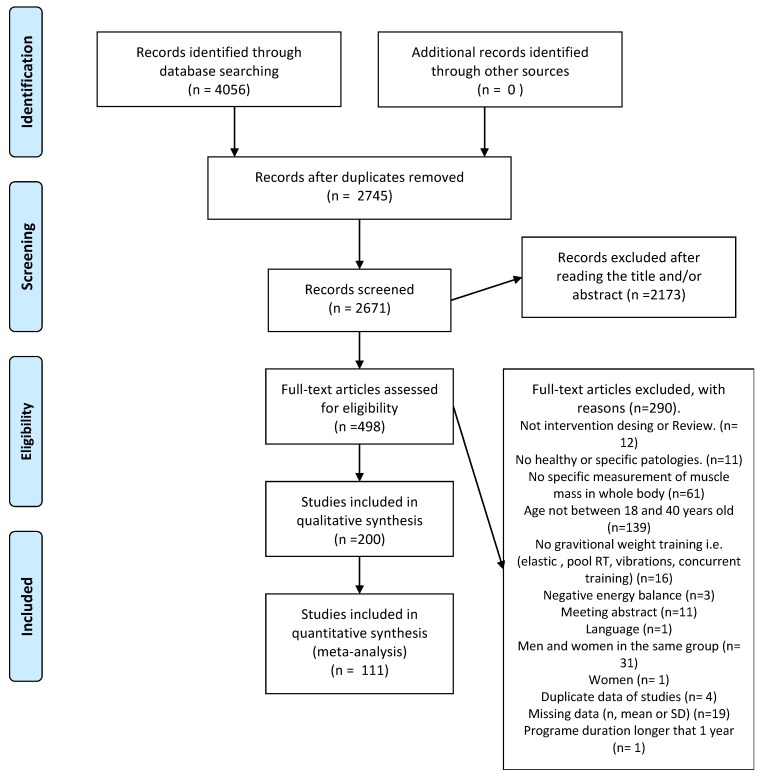
Prisma flow diagram for the included studies.

A comprehensive literature search was performed using PubMed-Medline, Web of Science and the Cochrane Library from database inception through to 10 May 2018. The database searches were performed independently by two authors (P.J.B. and D.J.R.-C.) and the results obtained were the same. The following combination terms was used: “muscle hypertrophy” or “muscle thickness” or “muscle gain” or “fat free mass” or “muscular size” or “muscle size” or “skeletal muscle mass” or “appendicular skeletal muscle mass” or “muscle mass” or “body fat free”. The Boolean operator “AND” was used to combine these descriptors with: “resistance training” or “strength training” or “weight training” or “power training” or “weightlifting” or “full body” or “circuit*” or “neuromuscular training” or “body weight training”. Also, the Boolean operator “NOT” was combined with the terms “sarcopenia” or “atroph*” or “Review”. The flow diagram of the search process is shown in Figure 1. 

### 2.3. Selection Criteria

The specific inclusion criteria were: (1) studies examining the effect of RT from for at least 2 weeks to one year on lean body mass, skeletal muscle mass or fat-free mass; (2) participants were men; (3) studies published in English, and; (4) studies provide information of outcomes both at baseline and follow-up. Research studies were excluded if they: (1) used a sample population with pathologies or not between 18 and 40 years of age; (2) used men and women in the same group of training; (3) performed RT with a negative energy balance; (4) were a review or did not have an intervention design; (5) were not an original investigation published in full; (6) did not specify the tests to be evaluated or did not use a specific measurement of muscle mass in whole body; (7) applied gravitational weight training (i.e., elastic bands, pool resistance training, concurrent training, body vibrations…); (8) did not provide or specify numerical data; and, (9) they examined acute effects of interventions. 

### 2.4. Study Selection and Data Extraction 

Retrieved articles were reviewed independently by two authors (P.J.B. and D.J.R.-C.), to choose potentially relevant articles; all disagreements on inclusion/exclusion were discussed and resolved by consensus. References of potentially relevant articles were also searched to find additional studies, and authors of selected studies were contacted for non-reported information. Two authors (P.J.B. and D.J.R.-C.) independently extracted data from the included studies. The following information was extracted: authors of the paper, study design, number of participants included in each group, training experience and training status, age, weight and height. Regarding the characteristics of the RT programmes, the information extracted included: the type of exercise (i.e., full body, circuit training, traditional split routine…), duration of training (weeks), training frequency (sessions/week), number of exercise performed each day, relative load lifted, sets and repetitions performed, rest between sets and between repetitions, total number of sessions, nutritional supplementation, drugs use and the outcomes measured (e.g., lean body mass, skeletal muscle mass or fat-free mass). 

### 2.5. Outcomes 

The output variables of the articles were defined as follows. Fat-free mass (FMM) was calculated as “all that is not fat”, subtracting fat weight from body weight, or when the measurements were obtained by dual X-ray absorptiometry was calculated as lean tissue plus bone mineral content [12]. Lean muscle mass (LMM), lean mass, lean body mass, bone-free lean body mass or mineral-free lean mass was calculated as the fat-free mass minus the bone mineral content (DXA) or as fat-free mass minus the estimated weight [13] of the live bone by the equation of Heymsfield et al. [14]. Skeletal muscle mass (SMM) or skeletal muscle was defined as lean muscle and was calculated by anthropometric equations, by proprietary algorithms when using bioimpedance or by estimates based on dual X-ray absorptiometry data [15].

### 2.6. Evaluation of the Methodology of the Studies Selected

The methodological quality of the selected studies was assessed with the quality assessment tool for before−after (pre−post) studies with no control group tool (National Heart Lung and Blood Institute, 2014), which analyses the following items: (1) study question; (2) eligibility criteria and study population; (3) study participants representative of clinical populations of interest; (4) all eligible participants enrolled; (5) sample size; (6) intervention clearly described; (7) outcome measures clearly described, valid, and reliable; (8) blinding of outcome assessors; (9) follow-up rate; (10) statistical analysis; (11) multiple outcome measures; and (12) group-level interventions and individual-level outcome efforts. For each study, each item was described as having either a low risk of bias, an unclear risk of bias or a high risk of bias. Risk of bias was assessed independently by two authors (P.J.B. and D.J.R.-C.) using the previously described risk of bias tool.

### 2.7. Data Synthesis and Statistical Analysis 

**Figure 2:**
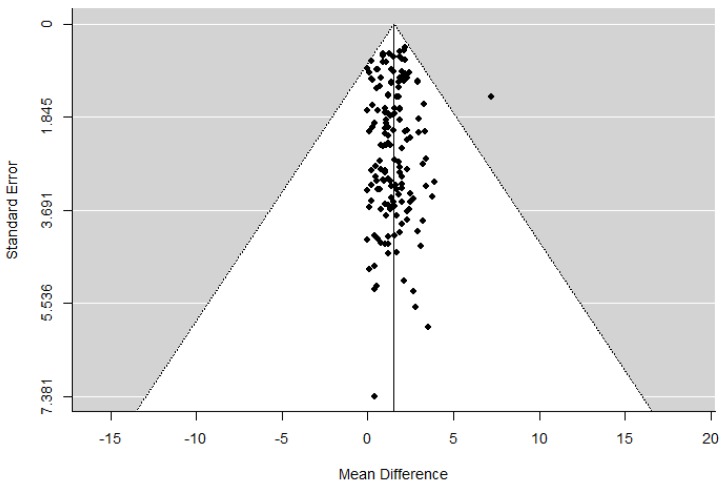
Test for funnel plot asymmetry of the change of muscle mass (all variables measured: FFM, LMM, SMM) after resistance training.

The meta-analysis and the statistical analysis were conducted using the Review Manager software (RevMan 5.2; Cochrane Collaboration, Oxford, UK). A random effects meta-analysis was conducted to determine the summary effect of RT on lean body mass, skeletal muscle mass or fat-free mass. The effects of training on these outcomes were expressed as mean differences (MD) and their 95% confidence intervals (CI). The heterogeneity between the studies was evaluated through the I^2^ statistic, and between-study variance using the tau-square (Tau2) [16]. The I^2^ values of 30−60% represented a moderate level of heterogeneity. A *p* value < 0.1 suggests the presence of substantial statistical heterogeneity. The publication bias was evaluated through an asymmetry test as estimated from a funnel plot (Figure 2). In addition, the Egger’s test was used to assess publication bias. A p-value of less than 0.05 was considered to be statistically significant. 

#### Effects of Moderator Variables: Meta-Regression and Sub-Analysis 

To explore the moderate effect related to the participants and characteristics of training, meta-regression and meta-analysis were performed. The continuous covariates were meta-regressed individually and together in a random-effects meta-regression model using Jamovi project (Package for R). The following prognostic factors were considered: average of age, weight, height, study durations (weeks), sessions, days per week, number of exercises per workout, rest between exercise, number of sets per workout, range repetitions, training duration (min) and average intensity (%1RM) and for training status the studies were coded according to the following structure: sedentary/untrained = 0; physically active but no experience in RT = 1; RT up to 1 year/intermediate = 2; RT experience up to 2 years/intermediate = 3; RT experience up to 3 years/intermediate = 4; RT experience of 4 years or more/advanced = 5. For the meta-regression, we used a residual restricted maximum likelihood to measure between-study variance (τ2). Factors found to be significant at *p* = 0.05 level were included in multivariate meta-regression models. In addition, the training status variable was considered to be a categorical variable and in order to do so the participants categorized as untrained were those described as untrained, without experience in RT or less than one year of experience with loads, and the participants with RT experience of more than one year were categorized as trained. Finally, subgroup analyses were used for the effects of/to find the effects of categorical variables (training status: untrained vs trained). 

## 3. Results

### 3.1. General Characteristics of Studies

The initial search, which was based on the effect of resistance training on muscle mass, identified 4056 articles from the databases and no articles from other sources. After removing the duplicates, 2671 abstracts were screened, 2173 were excluded and 498 were screened as full texts. Finally, 111 studies (see Supplementary Table S1) were determined to fulfil the inclusion criteria and thus selected for the meta-analysis (Figure 1). Publications ranged from 1973 to 2018. The instruments used to carry out the measurements were anthropometry (n groups = 19), ultrasound (n groups = 1), Bod Pod (n groups = 3), bioelectrical impedance analysis (BIA) (n groups = 14), dual-energy X-ray absorptiometry (DXA) (n groups = 35), hydrodensitometry, hydrostatic weighing and underwater weighing (n groups = 10) FFM; anthropometry (n groups = 4), BIA (n groups = 1), DXA (n groups = 48), underwater weighing (n groups = 12) for LMM and anthropometry (n groups = 8) and DXA (n groups = 3) for SMM. The main characteristics and properties of the included studies are summarized in Supplementary Table S1. 

#### 3.1.1. The Participants’ Characteristics

The initial search, which was based on the effect of resistance training on muscle mass, identified 111 articles (158 groups) and 1927 participants were measured (23.5 ± 3.31 years; 79.4 ± 6.42 kg and 177 ± 9.19 cm). Sixty-one studies (82 groups) analysed fat-free mass, 52 studies (65 groups) evaluated the lean muscle mass and seven studies (11 groups) examined the muscle mass to analyze the effects of a resistance training programme on muscle mass. Some of these studies measured more than one variable and the same study may be included in FFM, LMM or SMM (see Table 1). There were a total of 951 participants in fat-free mass (23.8 ± 3.40 years; 79.5 ± 6.36 kg and 178 ± 3.64 cm), 810 participants in lean body mass (23.2 ± 3.47 years; 80.4 ± 6.41 kg and 176 ± 1.40 cm) and 155 participants in muscle mass (23.0 ± 1.24 years; 73.5 ± 3.5 kg 179 ± 3.0 cm). The participants who carried out the intervention had a state of training between untrained to bodybuilder professionals; the frequency of each state was sedentary/untrained (n groups: FFM = 13; LMM = 3; SMM = 0); physically active without experience (n groups: FFM = 26; LMM = 13; SMM = 3); with experience in resistance training up to one year (n groups: FFM = 8; LMM = 14; SMM = 5); with experience 1−2 years (n groups: FFM = 8; LMM = 5; SMM = 0); with experience 2−4 years (n groups: FFM = 8; LMM = 6; SMM = 0); and with experience 4 years or more (n groups: FFM = 7; LMM = 0; SMM = 0).

#### 3.1.2. The Resistance Training Characteristics 

The types of training performed were circuit training (n groups: FFM = 1; LMM = 2), full body (n groups: FFM = 40; LMM = 31; SMM = 4), traditional split routine (n groups: FFM = 34; LMM = 20; SMM = 7), lower limbs (n groups: LMM = 3), and physical military training (n groups: FFM = 2) and not specified (n groups: FFM = 5; LMM = 9) for FFM. The training sessions lasted from 4 to 24 weeks (FFM = 10.4 ± 5.41; LMM = 9.37 ± 4.53; SMM = 10.5 ± 4.01) with a weekly frequency of between 2 and 6 days (FFM = 3.5 ± 0.84; LMM = 3.2 ± 0.81; SMM = 3.3 ± 0.94). The resistance training sessions were carried out with between 1 and 20 exercises (FFM = 7.9 ± 3.13; LMM = 7.3 ± 3.67; SMM = 7.0 ± 1.48), 1 to 50 sets per exercise (FFM = 16.7 ± 11.8; LMM = 14.0 ± 10.1; SMM = 19.1 ± 11.1) and 2 to 15 repetitions per set (FFM = 7.8 ± 3.0; LMM = 9.1 ± 1.9; SMM = 8.5 ± 0.7) with intensities of 50 and 89% of 1RM (FFM = 78.8 ± 5.8; LMM = 74.6 ± 8.6; SMM = 80.8 ± 5.8). 

### 3.2. Heterogeneity and Risk of Bias

Risk-of-bias assessment is shown in Figure 2. Overall, the risk of bias was low in the studies, the overall median scores were high, 10/12 points. 

In addition, heterogeneity was not found for changes in hypertrophy for overall effects (Z= −0.078; *p* = 0.94). In addition, heterogeneity was not observed when studies were divided according to FFM (Z= −0.003; *p* = 0.99); LMM (Z = −0.25, *p* = 0.80) and SMM (Z = −0.58, *p* = 0.56).

### 3.3. Meta-Analyses 

#### 3.3.1. Effects of Training on Hypertrophy

The results of the overall effects on muscle mass (FFM + LMM + SMM) before and after the resistance training programme showed significant improvement between pre- and post-test (n participants = 1916; 1.53 kg 95% CI [1.30, 1.76], *p* < 0.001; I^2^ = 0%, *p* = 1.00). 

#### 3.3.2. Subgroup Analysis

RT evoked a significant increase of FFM (1.56 kg 95% CI [1.23, 1.89]), of LMM (1.65 kg 95% CI [1.28, 2.01]) and of SMM (1.11 kg 95% CI [0.52, 1.71]) (Table 1). However, non-significant differences were observed between the different variables used to describe hypertrophy used.

**Figure 3:**
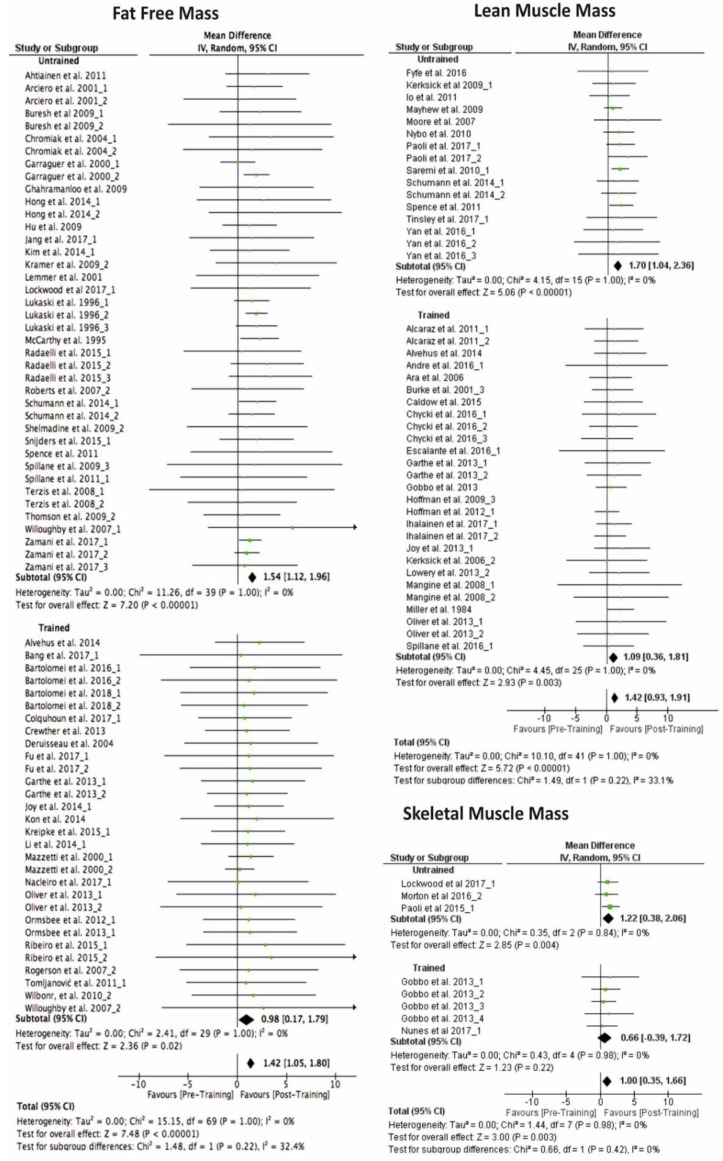
Mean differences exploration comparing the training effect.

When the studies were divided using the training status of participants, non- significant differences were observed between trained and untrained participants after the resistance training programme (Figure 3). However, when comparing pre−post intervention results for each way of measuring hypertrophy, both groups present significant increases for FFM (untrained: 1.54 kg 95% CI [1.12, 1.96], trained: 0.98 kg 95% CI [0.17, 1.79]), for LMM (untrained: 1.70 kg 95% CI [1.04, 2.36], trained: 1.09 kg 95% CI [0.36, 1.81]), except for the SMM (untrained: 1.22 kg 95% CI [0.38, 2.06], trained: 0.66 kg 95% CI [−0.39, 1.72]).

**Figure 4:**
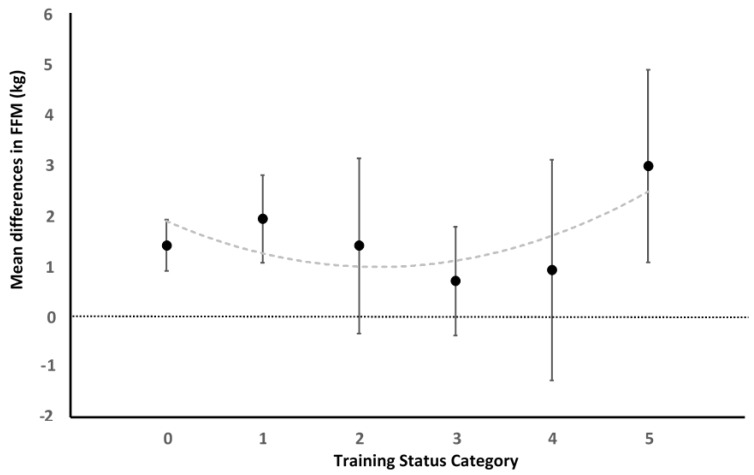
Mean differences exploration comparing the training status by categories. Sedentary = 0: 1.39 kg 95% CI [0.89, 1.90], *p* < 0.001; I^2^ = 0%, *p* = 0.89; untrained = 1: 1.92 kg 95% CI [1.05, 2.78], *p* < 0.001; I^2^ = 0%, *p* = 1.00; Up to one year experience = 2: 1.39 kg 95% CI [−0.35, 3.12], *p* = 0.12; I^2^ = 0%, *p* = 1.00; Up to two year experience = 3: 0.69 kg 95% CI [−0.39, 1.76], *p* =0.21; I^2^ = 0%, *p* = 1.00; Up to tree year experience = 4: 0.9 kg 95% CI [−1.28, 3.09], *p* = 0.42; I^2^ = 0%, *p* = 1.00; RT experience of 4 years or more = 5: 2.96 kg 95% CI [1.06, 4.87], *p* = 0.002; I^2^ = 41%, *p* = 0.08.

Performing a more detailed analysis of the training status on FFM, we found that the categories sedentary/untrained and physically active but no experience in RT, have higher and more significant values than the moderately trained (RT up to 1 year/intermediate, RT experience up to 2 years/intermediate, and RT experience up to 3 years/intermediate). Additionally, the highest values achieved are those obtained by the more experienced subjects, RT experience of 4 years or more/advanced (see Figure 4). However, no statistically significant differences between categories were observed (χ² = 5.79; *p* = 0.33). 

### 3.4. Meta-Regression

The results from the meta-regression model are presented in Table 2. Considering the overall effects, and taking into account the participant characteristics, none of our covariates explained any effect on changes in muscle mass. Regarding the training characteristics, the only significant variable that explains the variance of the hypertrophy was the sets per workout, showing a significant negative interaction (MD; estimate: 1.85, 95% CI [1.45, 2.25], *p* < 0.001; moderator: −0.03 95% CI [−0.05, −0.00] *p* = 0.041). When the effect of RT on changes in hypertrophy was evaluated with muscle mass assessment stratified into three subgroups (FFM, LMM and SMM) there were no covariates that explained any of the variance in the change in hypertrophy. 

## 4. Discussion

The main finding of the present study is that RT significantly increases muscle mass, in average (FFM = 1.56/LMM = 1.65/SMM = 1.11 kg), by about 1.5 kg with a wide range of heterogeneity (from 0 to 7.2 kg). These results were obtained including studies with interventions durations ranging from 2 weeks to one year. The present meta-analysis is, to date, the largest in terms of RT interventions on full body muscle mass gain. Most meta-analyses that have analysed the effect of RT and characteristics of participants on muscle mass gains have only included works with protein supplementation [6,122,123,124,125,126,127,128,129] or have analysed single variables of the training load isolated, as for example, training frequency [130,131,132], intensity or training periodization [133]. This segmentation has meant that many of the works that have focused solely on these analyses have been left out. However, as stated by Morton et al. [6], performance of RT alone is the much more potent stimulus, accounting for a substantially greater portion of the variance in RT-induced gain in muscle mass. Therefore, in order to have the largest number of studies that met all the inclusion criteria, the meta-analyses should focus on the variables of the training or the characteristics of the participants that have the greatest effect on muscle mass. The most important findings are that RT is an effective method to develop hypertrophy, regardless of the variable used to quantify it, as well as the training characteristics (intensity, volume, weekly training frequency, etc.), with significant increases of 1.6 kg in FFM; of 1.7 kg in LMM; and 1.1 kg in muscle mass (MM). These results are slightly higher than those found in the meta-regression of Morton et al. [6], increasing 1.1 kg of FFM when supplementation is not included and 1.4 kg in the FFM when supplementation is included. This may be due to the fact that only studies including intervention with nutritional supplements were considered in Morton’s work, and that the no-supplementation results were derived from the control groups of these studies. This considerably reduced their sample and may explain their possible underestimation of muscle mass. On the other hand, although there is no clear moderating factor, it is observed that there are certain variables such as the training status (the untrained have greater gains in hypertrophy), as well as other variables related to the training load, mainly the number of sets per workout, which explain a greater gain in this variable when the number is relatively low, always within a threshold. However, due to the lack of explanation of the variance from the variables of the training load analyzed, it has not been possible to fulfil the objective of creating a mathematical model for the estimation of the level of human muscle hypertrophy in men.

### 4.1. Participant Characteristics

Regarding the characteristics of the participants, no variable has been shown to moderate gains in hypertrophy. It should be noted that a decrease in hypertrophy has traditionally been associated with age. In the present meta-analysis, it has been shown that, regardless of the technique used to estimate muscle hypertrophy, age does not have a moderating component on it. These results are as expected, as the included studies focused only on participants between 18 and 40 years old. In humans, sarcopenia (i.e., loss of muscle mass and function) affects individuals from approximately the 4th decade of life [134], with a decrease of 30–50% in skeletal muscle mass and function by the time individuals reach approximately 80 years of age [135]. For instance, Morton et al.’s [6] study found that RT in addition of protein supplementation is more effective at improving FFM in young or resistance-trained individuals than in older or untrained individuals. It is clear that older individuals are anabolically resistant [136]; however, according to the current meta-analysis, it seems that RT induces similar gains in muscle mass, independently of the age of the participant, when considering individuals from 18 to 40 years old. In any case, future studies are needed in which it is analyzed whether the gains of muscle mass after RT are dependent on the age of the participants of said programmes, including in those studies that have a sample with a wide age range.

Furthermore, in relation to the initial level of the participants (training status) we observed the shorter the experience in RT of the athlete is, the higher the hypertrophy gains are, except for those with an experience of 4 years or more (Figure 4). We believe that the reasons for the similar improvements in sedentary and advanced subjects are due to different reasons. In the case of sedentary or lightly trained it is probably the lack of previous stimulus that causes greater hypertrophy. These results are in line with different studies that have shown that hypertrophic responses to RT have been shown to diminish over time [137], in addition to hypertrophic potential being lower in well-trained strength athletes [138]. However, regarding the most advanced practitioners our results are quite surprising. It seems that once an experience threshold of approximately 4 years is surpassed the ability to hypertrophy increase. We hypothesise that this might be due to a more advance knowledge about training methodologies, higher level of adherence to the RT sessions, greater motivation toward training [139], a better exercises technique or even or the use of undeclared anabolic steroids. However, more studies elucidating the factors (behavioural, physiological or other) responsible for this are needed. Finally, from these results we can recommend that if we want high gains in hypertrophy with intermediate trained participants, we will have to be very precise in the design and establishment of the appropriate training load, as well as in the nutritional strategies necessary for an optimal anabolic balance of athletes [6]. Therefore, the previous state of training is a decisive variable in the potential gain of muscle mass. If analysed as a dichotomous variable (trained/untrained, Figure 3), the result is different to that of making a more detailed analysis taking into account several categories of experience (Figure 4). In any case, the relationship between hypertrophy and the training status does not seem linear. It should also be remembered that one possible explanation for this is that there are not enough studies with intermediate training states. Finally, only men were included in the present study because the ability to hypertrophy is very different depending on the sex [10]. In addition, several studies suggest that the menstrual cycle can influence muscle mass improvements [140,141].

### 4.2. Training Characteristics

With respect to the training characteristics the only variable that seems to have a significant moderating effect is the number of series per session, being its moderator coefficient negative (−0.03; *p* = 0.04). This suggests that a high number of series per session could negatively affect muscle mass gains. This finding is in line with those by Schoenfeld et al. [130,132] showing that when equaling volume, greater hypertrophy was observed in those groups that distributed the sets with a higher weekly frequency (2−3 days) per muscle group. Contrary to these results (ours and previous) there is a meta-analysis supporting the efficacy of greater exercise volume [142], concluding that individuals interested in achieving maximal hypertrophy should perform a minimum of 2–3 sets per exercise, and that possibly 4–6 sets could give even a greater response; yet still, considerable heterogeneity was present in the analysis [143], and the only significant difference was observed when comparing one set with three sets. Nevertheless, even if a minimum number of series seems to be necessary to maximise muscle hypertrophy [144] there also appears to be a threshold by which the increase in the sets of exercise performed per muscle group within a given training session does not necessarily lead to greater muscle growth [144,145]. Thus, when designing a RT programme aiming to increase muscle mass it is not recommended to include an excessively high number of sets, such as that found in this study (16 sets per session on average). Similar recommendations have recently been proposed in a narrative review suggesting that despite increasing the number sets per exercise (albeit the majority of studies within RT literature focus on number of sets), it is likely more beneficial to increase the training frequency [145].

Therefore, future analyses are necessary in which all the variables related to the training load are stratified so that they can give us an idea of which is the optimal dose. Consequently, although we observed an influence of number of sets over the quantity of muscle hypertrophy developed, more studies are needed.

However, perhaps the methods used so far for the muscle hypertrophy may not be sensitive enough to predict which variable and to what extent said variable can moderate greater gains in the development of hypertrophy.

## 5. Conclusions

In conclusion, resistance training has a significant effect on the improvement of hypertrophy, regardless of the method used to quantify it. The improvements ranged from 1.6 kg in FFM, 1.7 kg in LMM, to 1.1 kg in MM. Regarding the characteristics of the participants, there are no variables (neither the age, nor the training status of the participants) that moderate the gains in hypertrophy. In addition, with respect to the characteristics of the training, the only single variable that moderates inversely the gains in hypertrophy is the number of sets per workout, showing that an excess of sets per workouts affects negatively the amount of muscle growth. 

## 6. Limitations

As mentioned earlier, RT has a long and significant effect on the development of hypertrophy. However, these gains were different depending on the variable used as the equivalent of muscle hypertrophy. Clearly, the methodologies used differ from each other, finding a non-significant difference of 45.6−54.6% when comparing hypertrophy by means of FFM and LMM versus MM. In the context of body composition and muscle hypertrophy, it is important to be very clear about what these two concepts mean and how they are measured as variables. Ideally, muscle mass should be measured isolated from the rest of the tissues, but this can only be achieved by using nuclear magnetic resonance and most of the studies do not utilize this method. Therefore, very few studies assess muscle mass as an isolated variable from the rest of the tissues and/or provide the value for the whole-body muscle mass [13,15,146].

The methodology of assessment could be a limitation, given that the measurement techniques (i.e., DXA, bioimpedance, etc.) of each study are different. However, previous studies have reported that these differences are not significant [147], supporting the comparison made in the present work.

In addition, we need to keep in mind that many of the included studies did not specifically aimed to increase muscle mass, and therefore the heterogeneity of the results may be due to the different methodologies employed. Nevertheless, the purpose of our study was to define the range of muscle mass growths that can be expected after a RT intervention, even if the main purpose of the programmes was not muscle hypertrophy.

Finally, the lack of heterogeneity in the variance of the studies include in the present meta-analysis, the different purposes and outcomes expected by the studies included, and poor description of training variables may be limiting to the present meta-analysis and meta-regression.

## Figures and Tables

**Table 1:** Main differences between pre-post study in the main body composition variable considered. The numerical suffix after the references indicate the different subgroup in the same study. See supplemental material for more information.

Study/Group/Year/Reference	Post-Training			Pre-Training			Weight	Mean Difference
	Mean	SD	n	Mean	SD	n		Random, 95% IC
Fat Free Mass (FFM)								
Abe et al., 2000 [17]	62.7	5.3	17	61.1	5.1	17	0.90%	1.60 [−1.90, 5.10]
Ahtiainen et al., 2011 [18]	68.0	4.1	7	65.0	4.0	7	0.60%	3.00 [−1.24, 7.24]
Álvarez, et al., 2012_1 [19]	60.0	6.6	5	56.9	7.3	5	0.10%	3.10 [−5.53, 11.73]
Álvarez, et al., 2012_2 [19]	59.2	6.8	5	58.2	7.0	5	0.20%	1.00 [−7.55, 9.55]
Alvehus et al., 2014 [20]	65.5	7.0	17	63.2	6.4	17	0.50%	2.30 [−2.21, 6.81]
Arazi et al., 2015_1 [21]	63.6	6.5	10	61.7	6.2	10	0.40%	1.90 [−3.67, 7.47]
Arciero et al., 2001_1 [22]	64.7	3.6	10	63.0	2.8	10	1.40%	1.70 [−1.13, 4.53]
Arciero et al., 2001_2 [22]	62.5	6.5	10	62.3	6.5	10	0.30%	0.20 [−5.50, 5.90]
Bang et al., 2017_1 [23]	70.9	10.2	8	70.5	10.8	8	0.10%	0.40 [−9.89, 10.69]
Bartolomei et al., 2016_1 [24]	73.8	7.5	10	72.0	7.6	10	0.30%	1.80 [−4.82, 8.42]
Bartolomei et al., 2016_2 [24]	70.6	9.4	8	69.4	8.8	8	0.10%	1.21 [−7.71, 10.13]
Bartolomei et al., 2018_1 [12]	75.4	7.5	9	73.7	8.6	9	0.20%	1.70 [−5.76, 9.16]
Bartolomei et al., 2018_2 [12]	73.5	7.7	11	72.8	7.7	11	0.30%	0.70 [−5.74, 7.14]
Bhasin et al., 1996_1 [25]	74.1	2.2	9	72.1	2.3	9	2.50%	2.00 [−0.08, 4.08]
Buresh et al., 2009_1 [26]	74.7	3.7	6	72.4	3.6	6	0.60%	2.30 [−1.83, 6.43]
Buresh et al., 2009_2 [26]	65.6	7.9	6	64.4	6.7	6	0.20%	1.20 [−7.09, 9.49]
Chromiak et al., 2004_1 [27]	71.4	9.5	18	68.0	9.8	18	0.30%	3.40 [−2.91, 9.71]
Chromiak et al., 2004_2 [27]	68.3	9.9	15	66.8	9.8	15	0.20%	1.50 [−5.55, 8.55]
Colquhoun et al., 2017_1 [28]	72.2	5.4	11	71.4	5.9	11	0.50%	0.80 [−3.93, 5.53]
Crewther et al., 2013 [29]	77.7	5.2	12	76.7	5.5	12	0.60%	1.00 [−3.28, 5.28]
Deruisseau et al., 2004 [30]	64.5	8.6	13	63.1	9.0	13	0.20%	1.40 [−5.37, 8.17]
Fu et al., 2017_1 [31]	63.4	9.8	9	62.2	8.7	9	0.10%	1.19 [−7.37, 9.75]
Fu et al., 2017_2 [31]	62.8	7.7	9	61.5	7.8	9	0.20%	1.35 [−5.85, 8.55]
Gallagher et al., 2000_1 [32]	65.3	2.2	14	65.3	2.5	14	3.60%	0.00 [−1.74, 1.74]
Gallagher et al., 2000_2 [32]	66.3	1.6	12	64.4	1.6	12	6.70%	1.90 [0.62, 3.18]
Garthe et al., 2013_1 [33]	65.0	8.7	21	63.4	8.7	21	0.40%	1.60 [−3.67, 6.87]
Garthe et al., 2013_2 [33]	65.6	6.2	18	64.6	6.3	18	0.70%	1.00 [−3.07, 5.07]
Ghahramanloo et al., 2009 [34]	57.1	6.1	9	55.2	6.4	9	0.30%	1.90 [−3.88, 7.68]
Hong et al., 2014_1 [35]	64.7	7.6	8	62.0	6.3	8	0.20%	2.66 [−4.15, 9.47]
Hong et al., 2014_2 [35]	63.8	8.1	10	60.0	7.2	10	0.20%	3.80 [−2.93, 10.53]
Hu et al., 2009 [36]	66.5	6.7	48	65.3	7.0	48	1.50%	1.20 [−1.54, 3.94]
Huso et al., 2002_2 [37]	65.4	1.8	10	63.2	1.4	10	5.50%	2.20 [0.79, 3.61]
Jang et al., 2017_1 [38]	60.3	6.2	8	58.9	6.7	8	0.30%	1.40 [−4.93, 7.73]
Joy et al., 2014_1 [39]	60.7	4.7	14	59.5	4.7	14	0.90%	1.20 [−2.29, 4.69]
Kim et al., 2014 [40]	52.7	4.1	9	51.5	4.6	9	0.70%	1.20 [−2.83, 5.23]
Kon et al., 2014 [41]	55.8	7.9	7	53.8	6.9	7	0.20%	2.00 [−5.78, 9.78]
Kramer et al., 2009_2 [42]	61.6	6.8	9	57.7	6.5	9	0.30%	3.90 [−2.23, 10.03]
Kreipke et al., 2015_1 [43]	62.9	4.9	13	61.8	4.8	13	0.80%	1.10 [−2.63, 4.83]
Lemmer et al., 2001 [44]	64.9	7.1	10	62.9	7.1	10	0.30%	2.00 [−4.23, 8.23]
Li et al., 2014 [45]	71.7	2.8	13	70.6	8.2	13	0.50%	1.04 [−3.69, 5.77]
Lockwood et al., 2017_1 [46]	61.7	8.5	15	60.4	8.5	15	0.30%	1.30 [−4.79, 7.39]
Lukaski et al., 1996_1 [47]	67.3	2.2	12	65.9	2.2	12	3.50%	1.40 [−0.36, 3.16]
Lukaski et al., 1996_2 [47]	65.9	1.5	12	64.0	1.2	12	9.30%	1.90 [0.81, 2.99]
Lukaski et al., 1996_3 [47]	66.1	2.6	12	64.2	2.6	12	2.50%	1.90 [−0.18, 3.98]
Mazzetti et al., 2000_1 [48]	69.6	2.6	10	68.2	2.6	10	2.10%	1.38 [−0.89, 3.65]
Mazzetti et al., 2000_2 [48]	68.4	1.5	8	68.2	1.5	8	5.20%	0.25 [−1.20, 1.70]
McCarthy et al., 1995 [49]	68.1	2.3	10	65.9	2.1	10	3.00%	2.20 [0.27, 4.13]
Nacleiro et al., 2017_1 [50]	64.2	7.3	8	64.2	7.3	8	0.20%	0.08 [−7.05, 7.21]
Noonan et al., 1998_1 [51]	88.3	10.4	13	85.1	9.5	13	0.20%	3.20 [−4.46, 10.86]
Oliver et al., 2013_1 [52]	71.9	9.8	11	70.0	9.6	11	0.20%	1.90 [−6.21, 10.01]
Oliver et al., 2013_2 [52]	72.3	9.9	11	71.9	9.8	11	0.20%	0.40 [−7.83, 8.63]
Ormsbee et al., 2012_1 [53]	64.7	5.9	11	63.5	5.2	11	0.50%	1.20 [−3.45, 5.85]
Ormsbee et al., 2013_1 [54]	68.2	6.0	11	66.9	5.3	11	0.50%	1.30 [−3.43, 6.03]
Piirainen et al., 2008_1 [55]	65.9	6.6	6	65.3	8.1	6	0.20%	0.60 [−7.76, 8.96]
Piirainen et al., 2008_2 [55]	64.3	9.4	6	63.8	8.6	6	0.10%	0.50 [−9.69, 10.69]
Pérez-Gómez et al., 2013 [56]	59.7	7.7	8	58.6	7.5	8	0.20%	1.09 [−6.35, 8.53]
Radaelli et al., 2015_1 [57]	67.7	6.5	12	67.2	8.3	12	0.30%	0.46 [−5.49, 6.41]
Radaelli et al., 2015_2 [57]	66.0	5.2	13	63.0	4.4	13	0.80%	2.98 [−0.71, 6.67]
Radaelli et al., 2015_3 [57]	74.7	5.0	13	71.4	5.9	13	0.60%	3.32 [−0.89, 7.53]
Ribeiro et al., 2015_1 [58]	75.0	6.4	5	72.1	6.6	5	0.20%	2.90 [−5.16, 10.96]
Ribeiro et al., 2015_2 [58]	76.5	9.9	5	73.0	9.1	5	0.10%	3.50 [−8.29, 15.29]
Roberts et al., 2007_2 [59]	64.9	8.0	16	63.9	8.4	16	0.30%	1.00 [−4.68, 6.68]
Rogerson et al., 2007_2 [60]	78.0	8.4	11	76.8	8.4	11	0.20%	1.20 [−5.82, 8.22]
Schumann et al., 2014_1 [61]	62.9	2.6	16	60.9	2.8	16	3.10%	2.00 [0.13, 3.87]
Schumann et al., 2014_2 [61]	60.7	3.6	18	59.3	3.5	18	2.00%	1.40 [−0.92, 3.72]
Shelmadine et al., 2009_2 [62]	55.8	6.8	9	54.9	6.4	9	0.30%	0.95 [−5.16, 7.06]
Snijders et al., 2015_1 [63]	64.8	6.1	19	62.9	5.7	19	0.80%	1.90 [−1.85, 5.65]
Spence et al., 2011 [64]	65.5	9.7	13	63.1	9.0	13	0.20%	2.40 [−4.79, 9.59]
Spillane et al., 2009_3 [65]	56.3	10.2	10	54.6	10.1	10	0.10%	1.70 [−7.18, 10.58]
Spillane et al., 2011_1 [66]	57.0	9.9	19	56.4	10.3	19	0.30%	0.59 [−5.83, 7.01]
Terzis et al., 2008 [67]	65.4	9.6	8	65.0	9.6	8	0.10%	0.40 [−9.01, 9.81]
Terzis et al., 2008b [68]	62.6	6.6	11	62.2	6.6	11	0.40%	0.44 [−5.08, 5.96]
Thomson et al., 2009_2 [69]	61.8	5.4	17	61.4	6.1	17	0.70%	0.40 [−3.47, 4.27]
Tomljanović et al., 2011 [70]	69.2	10.4	23	68.3	10.6	23	0.30%	0.92 [−5.15, 6.99]
Wilborn, et al., 2010_2 [71]	69.6	8.1	13	67.9	8.3	13	0.30%	1.64 [−4.64, 7.92]
Willoughby et al., 2007_2 [72]	68.8	10.3	10	63.2	9.4	10	0.10%	5.62 [−3.02, 14.26]
Willoughby et al., 2014_1 [73]	63.9	11.9	9	61.2	10.6	9	0.10%	2.70 [−7.69, 13.09]
Wilson et al., 2013_1 [74]	70.5	7.6	10	68.5	8.2	10	0.20%	2.00 [−4.93, 8.93]
Wilson et al., 2014_1 [75]	69.2	1.1	9	67.1	1.1	9	10.60%	2.10 [1.08, 3.12]
Zamani et al., 2017_1 [76]	56.2	1.3	10	54.9	1.3	10	8.30%	1.26 [0.11, 2.41]
Zamani et al., 2017_2 [76]	54.6	1.6	10	53.7	1.2	10	6.90%	0.91 [−0.35, 2.17]
Zamani et al., 2017_3 [76]	50.5	5.9	10	49.8	6.3	10	0.40%	0.72 [−4.63, 6.07]
Total (95% CI)			951			951	100.00%	1.56 [1.23, 1.89]
Heterogeneity: Tau² = 0.00; Chi² = 18.14, df = 81 (*p* = 1.00); I² = 0%					Test for overall effect: Z = 9.22 (*p* < 0.00001)			
Lean Muscle Mass (LMM)								
Alcaraz et al., 2011_1 [77]	56.4	5.3	11	55.2	5.9	11	0.5%	1.20 [−3.49, 5.89]
Alcaraz et al., 2011_2 [77]	60.3	5.2	15	58.8	4.6	15	1.0%	1.50 [−2.01, 5.01]
Alvehus et al., 2014 [20]	59.0	6.5	17	56.8	6.0	17	0.7%	2.20 [−2.00, 6.40]
Andre et al., 2016_1 [78]	60.5	9.1	10	58.9	9.7	10	0.2%	1.60 [−6.64, 9.84]
Ara et al., 2006 [79]	56.5	4.2	12	55.9	4.2	12	1.1%	0.60 [−2.76, 3.96]
Bemben et al., 2001_2 [80]	95.9	6.7	8	95.7	7.3	8	0.3%	0.20 [−6.67, 7.07]
Brown et al., 1999_2 [81]	66.0	2.5	10	63.1	2.6	10	1.6%	2.90 [0.66, 5.14]
Burke et al., 2001_3 [82]	62.5	2.6	5	61.5	2.7	5	0.8%	1.00 [−2.29, 4.29]
Caldow et al., 2015 [83]	60.7	5.6	10	59.6	5.2	10	0.5%	1.10 [−3.64, 5.84]
Chycki et al., 2016_1 [84]	60.6	5.2	6	58.6	5.3	6	0.3%	2.00 [−3.94, 7.94]
Chycki et al., 2016_2 [84]	64.3	3.2	6	63.2	3.6	6	0.8%	1.10 [−2.75, 4.95]
Chycki et al., 2016_3 [84]	63.2	3.6	6	63.1	3.8	6	0.7%	0.10 [−4.09, 4.29]
Deyssig et al., 1993_1 [85]	83.6	3.2	11	76.4	3.6	11	1.5%	7.20 [4.35, 10.05]
Escalante et al., 2016_1 [86]	62.0	9.7	10	61.2	9.7	10	0.2%	0.80 [−7.70, 9.30]
Fahey and Brown 1973_1 [87]	66.1	9.4	13	63.6	8.6	13	0.2%	2.50 [−4.43, 9.43]
Falk et al., 2003_1 [88]	79.0	9.2	15	77.0	8.6	15	0.3%	2.00 [−4.37, 8.37]
Falk et al., 2003_2 [88]	72.7	7.7	13	71.5	8.0	13	0.3%	1.20 [−4.84, 7.24]
Fyfe et al., 2016 [89]	60.9	5.5	8	60.1	6.0	8	0.4%	0.80 [−4.84, 6.44]
Garthe et al., 2013_1 [33]	61.2	8.8	21	59.4	8.9	21	0.4%	1.80 [−3.55, 7.15]
Garthe et al., 2013_2 [33]	61.1	6.6	18	59.9	6.7	18	0.6%	1.20 [−3.14, 5.54]
Glowacki et al., 2004 [90]	64.3	8.5	13	61.8	8.7	13	0.3%	2.50 [−4.11, 9.11]
Gobbo et al., 2013 [91]	32.4	3.5	15	31.7	3.3	15	2.0%	0.70 [−1.73, 3.13]
Harber et al., 2004 [92]	67.3	3.4	8	65.4	3.4	8	1.1%	1.90 [−1.43, 5.23]
Hoffman et al., 2009_3 [93]	77.0	14.3	7	76.6	13.3	7	0.1%	0.40 [−14.07, 14.87]
Hoffman et al., 2012 [94]	65.6	2.2	9	65.5	1.9	9	3.3%	0.10 [−1.80, 2.00]
Ihalainen et al., 2018_1 [95]	62.5	6.1	37	61.3	6.1	37	1.5%	1.20 [−1.58, 3.98]
Ihalainen et al., 2018_2 [95]	59.1	5.0	31	58.6	5.1	31	1.9%	0.50 [−2.01, 3.01]
Joy et al., 2014_1 [39]	61.0	5.6	12	58.5	5.5	12	0.6%	2.50 [-1.94, 6.94]
Joy et al., 2016_1 [96]	65.5	6.9	11	65.3	8.1	11	0.3%	0.20 [−6.09, 6.49]
Kelly et al, 1998_1 [97]	78.0	11.8	9	75.2	12.0	9	0.1%	2.80 [−8.20, 13.80]
Kerksick et al., 2009_1 [98]	62.2	6.1	24	61.2	6.1	24	1.0%	1.00 [−2.45, 4.45]
Kerksick et al., 2006_3 [99]	63.5	7.3	11	63.5	8.2	11	0.3%	0.00 [−6.49, 6.49]
King et al., 1999_1 [100]	64.1	2.4	9	61.2	2.5	9	2.3%	2.90 [0.64, 5.16]
Ko and Choi 2013 [101]	56.0	10.9	18	55.0	10.5	18	0.2%	1.00 [−5.99, 7.99]
Kreider et al., 2002_1 [102]	76.1	3.0	23	75.5	3.1	23	3.8%	0.60 [−1.16, 2.36]
Lemon et al., 1992_2 [103]	73.4	10.5	12	73.4	10.5	12	0.2%	0.00 [−8.40, 8.40]
lo et al., 2011 [104]	50.8	3.3	10	50.5	3.9	10	1.2%	0.30 [−2.87, 3.47]
Lowery et al., 2014 [105]	60.8	5.8	12	58.8	6.3	12	0.5%	2.00 [−2.85, 6.85]
Mangine et al., 2008_1 [106]	71.4	9.9	8	69.3	10.5	8	0.1%	2.10 [−7.90, 12.10]
Mangine et al., 2008_2 [106]	71.8	8.4	9	69.5	8.1	9	0.2%	2.30 [−5.32, 9.92]
Mayhew et al., 2009 [107]	50.5	2.5	21	49.6	2.4	21	5.4%	0.90 [−0.58, 2.38]
Miller et al., 1984 [108]	66.6	2.2	8	64.3	2.1	8	2.7%	2.30 [0.19, 4.41]
Moore et al., 2007 [109]	64.8	6.7	12	61.6	6.9	12	0.4%	3.20 [−2.24, 8.64]
Nybo et al., 2010 [9]	62.8	2.7	8	61.0	2.3	8	2.0%	1.80 [−0.66, 4.26]
Oliver et al., 2013_1 [52]	64.2	8.5	11	61.9	8.9	11	0.2%	2.30 [−4.97, 9.57]
Oliver et al., 2013_2 [52]	64.3	6.8	11	63.3	7.0	11	0.4%	1.00 [−4.77, 6.77]
Paoli et al., 2017_1 [110]	62.4	3.3	18	60.3	3.5	18	2.4%	2.10 [−0.12, 4.32]
Paoli et al., 2017_2 [110]	64.2	4.9	18	60.9	4.7	18	1.2%	3.30 [0.16, 6.44]
Peeters et al., 1999_1 [111]	75.4	12.9	14	75.3	12.8	14	0.1%	0.10 [−9.42, 9.62]
Peronnet et al., 1986 [112]	66.3	2.1	7	64.5	2.2	7	2.4%	1.80 [−0.45, 4.05]
Pérez-Gómez et al., 2013 [56]	58.3	7.1	8	57.5	7.6	8	0.2%	0.80 [−6.41, 8.01]
Saremi et al., 2010_1 [113]	62.3	1.3	8	60.3	1.5	8	6.3%	2.00 [0.62, 3.38]
Schneider et al., 2003 [114]	59.1	3.3	7	57.2	3.0	7	1.1%	1.90 [−1.40, 5.20]
Schumann et al., 2014_1 [61]	57.2	5.0	16	55.6	4.5	16	1.1%	1.60 [−1.70, 4.90]
Schumann et al., 2014_2 [61]	55.9	4.6	18	54.1	4.1	18	1.5%	1.80 [−1.05, 4.65]
Slater et al., 2001_1 [115]	70.1	1.0	7	69.2	1.2	7	8.9%	0.90 [−0.26, 2.06]
Spence et al., 2011 [64]	59.1	2.5	13	56.9	2.5	13	3.2%	2.20 [0.28, 4.12]
Spillane et al., 2016_1 [116]	62.1	5.3	11	61.8	4.3	11	0.7%	0.30 [−3.73, 4.33]
Taylor et al., 2011_1 [117]	66.2	8.3	15	65.7	8.8	15	0.3%	0.50 [−5.62, 6.62]
Thorstensson et al., 1976 [118]	65.0	1.2	14	62.8	1.3	14	13.9%	2.20 [1.27, 3.13]
Tinsley et al., 2017_1 [119]	58.7	8.0	18	56.4	9.3	18	0.4%	2.30 [−3.37, 7.97]
Volek et al., 2004 [120]	70.6	5.8	9	67.2	5.6	9	0.4%	3.40 [−1.87, 8.67]
Yan et al., 2016_1 [121]	58.8	4.7	8	57.9	5.0	8	0.5%	0.90 [−3.86, 5.66]
Yan et al., 2016_2 [121]	61.0	7.7	9	59.5	7.3	9	0.2%	1.50 [−5.43, 8.43]
Yan et al., 2016_3 [121]	59.3	6.6	8	57.6	6.5	8	0.3%	1.70 [−4.72, 8.12]
Total (95% CI)			810			810	100%	1.65 [1.28, 2.01]
Heterogeneity: Tau² = 0.00; Chi² = 33.79, df = 64 (*p* = 1.00); I² = 0%					Test for overall effect: Z = 8.91 (*p* < 0.00001)			

**Table 2:** Metaregression analisys taking in to account de participants and training characteristics, at the begin the study.

Model	n Studies/ Groups	Estimate	Lower	Upper	*p*	Estimate	Lower	Upper	*p*	τ^2^	Adj. R^2^	I^2^	*p*
		Intrcpt				Moderator coeff.				Heterogeneity			
Overall Effects													
No covariables	158	1.530	1.300	1.753	<0.001					0 (SE = 0.13)		0%	1.000
Participants Characteristics													
Age (years)	142	0.943	−0.962	2.849	0.332	0.02	−0.06	0.10	0.58	0 (SE = 0.15)	NA%	0%	1.000
Weight (kg)	150	1.138	−1.822	4.098	0.451	0.00	−0.03	0.04	0.82	0 (SE = 0.14)	NA%	0%	1.000
Height (m)	126	−0.014	−6.032	6.004	0.996	0.80	−2.58	4.18	0.64	0 (SE = 0.15)	NA%	0%	1.000
Training Status	119	1.547	1.167	1.927	<0.001	−0.16	−0.40	0.08	0.19	0 (SE = 0.18)	NA%	0%	1.000
Training Characteristics													
Study Durations (weeks)	156	1.428	0.896	1.960	<0.001	0.01	−0.04	0.06	0.69	0 (SE = 0.13)	NA%	0%	1.000
Sessions	152	1.492	0.939	2.045	<0.001	0.00	−0.02	0.02	0.89	0 (SE = 0.13)	NA%	0%	1.000
Days per week	149	1.528	0.502	2.553	0.004	−0.01	−0.31	0.30	0.97	0 (SE = 0.14)	NA%	0%	1.000
n exercises per workout	141	1.423	0.640	2.207	<0.001	0.01	−0.09	0.11	0.87	0 (SE = 0.16)	NA%	0%	1.000
Rest between exercise (min)	121	1.336	0.646	2.025	<0.001	0.05	−0.23	0.32	0.74	0 (SE = 0.16)	NA%	0%	1.000
n set per workout	123	1.853	1.453	2.253	<0.001	−0.03	−0.05	−0.00	0.04	0 (SE = 0.14)	NA%	0%	1.000
range repetitions	123	1.257	0.486	2.029	0.001	0.02	−0.07	0.12	0.61	0 (SE = 0.17)	NA%	0%	1.000
Training duration (min)	35	1.548	−0.248	3.344	0.091	0.00	−0.03	0.03	0.75	0 (SE = 0.40)	NA%	0%	1.000
Average Intensity (%1RM)	89	2.353	−1.383	6.089	0.217	−0.01	−0.06	0.04	0.63	0 (SE = 0.22)	NA%	0%	1.000
Fat Free Mass													
No covariables	82	1.550	1.223	1.886	<0.001					0 (SE = 1.91)		0%	1.000
Participants Characteristics													
Age (years)	77	1.116	−1.534	3.766	0.409	0.02	−0.09	0.13	0.74	0 (SE = 1.95)	NA%	0%	1.000
Weight (kg)	80	−0.183	−4.371	4.006	0.932	0.02	−0.03	0.08	0.42	0 (SE = 0.21)	NA%	0%	1.000
Height (m)	67	−1.960	−18.842	14.930	0.82	1.96	−7.55	11.46	0.69	0 (SE = 0.21)	NA%	0%	1.000
Training Status	70	1.530	1.082	1.978	<0.001	−0.13	−0.41	0.15	0.37	0 (SE = 0.24)	NA%	0%	1.000
Training Characteristics													
Study Durations (weeks)	80	1.314	0.534	2.094	<0.001	0.02	−0.05	0.10	0.50	0 (SE = 0.20)	NA%	0%	1.000
Sessions	76	1.172	0.297	2.047	0.009	0.01	−0.01	0.04	0.35	0 (SE = 0.20)	NA%	0%	1.000
Days per week	76	1.154	−0.325	2.633	0.126	0.11	−0.30	0.53	0.59	0 (SE = 0.20)	NA%	0%	1.000
n exercises per workout	73	1.280	0.238	2.321	0.016	0.04	−0.10	0.17	0.59	0 (SE = 0.21)	NA%	0%	1.000
Rest between exercise (min)	56	2.086	0.096	4.075	0.04	-0.36	−1.40	0.68	0.50	0 (SE = 0.27)	NA%	0%	1.000
n set per workout	66	1.739	1.181	2.296	<0.001	-0.01	−0.05	0.02	0.49	0 (SE = 0.20)	NA%	0%	1.000
range repetitions	72	1.199	0.335	2.064	0.007	0.05	−0.07	0.17	0.38	0 (SE = 0.20)	NA%	0%	1.000
Training duration (min)	26	1.467	−1.415	4.349	0.318	0.01	−0.05	0.06	0.79	0 (SE = 0.47)	NA%	0%	1.000
Average Intensity (%1RM)	45	3.993	−3.027	11.012	0.265	-0.03	−0.12	0.06	0.48	0 (SE = 0.26)	NA%	0%	1.000
Lean Muscle Mass													
No covariables	65	1.644	1.275	2.013	<0.0001					0.03 (SE = 0.24)		1%	0.999
Participants Characteristics													
Age (years)	54	1.020	−1.964	4.005	0.5028	0.02	−0.10	0.14	0.73	0.03 (SE = 0.33)	NA%	1%	0.996
Weight (kg)	60	4.870	0.002	9.739	0.0499	−0.04	−0.10	0.02	0.18	0 (SE = 0.25)	NA%	0%	1.000
Height (m)	49	0.107	−6.453	6.666	0.9746	0.73	−2.94	4.40	0.70	0 (SE = 0.33)	NA%	0%	1.000
Training Status	41	1.584	0.721	2.446	0.0003	−0.15	−0.66	0.36	0.57	0 (SE = 0.44)	NA%	0%	1.000
Training Characteristics													
Study Durations (weeks)	65	1.701	0.881	2.521	<0.0001	−0.01	−0.08	0.07	0.87	0.04 (SE = 0.26)	0.00	2%	0.999
Sessions	65	1.860	1.087	2.633	<0.0001	−0.01	−0.03	0.02	0.53	0.05 (SE = 0.26)	0	2%	0.999
Days per week	62	1.996	−0.184	4.175	0.0727	--0.13	−0.84	0.58	0.72	0 (SE = 0.24)	NA%	0%	1.000
n exercises per workout	57	1.661	0.289	3.032	0.0176	−0.01	−0.18	0.16	0.92	0 (SE = 0.45)	NA%	0%	1.000
Rest between exercise (min)	54	1.458	0.279	2.636	0.0153	0.06	−0.37	0.50	0.78	0 (SE = 0.26)	NA%	0%	1.000
n set per workout	46	2.082	1.417	2.748	<0.0001	−0.04	−0.09	0.01	0.09	0 (SE = 0.29)	NA%	0%	1.000
range repetitions	44	0.601	−1.874	3.076	0.6341	0.08	−0.17	0.33	0.54	0 (SE = 0.43)	NA%	0%	1.000
Training duration (min)	9	1.256	−1.867	4.379	0.4306	0.01	−0.03	0.05	0.75	0 (SE = 1.61)	NA%	0%	0.998
Average Intensity (%1RM)	41	0.807	−3.984	5.598	0.7413	0.01	−0.05	0.07	0.77	0 (SE = 0.54)	NA%	0%	1.000
Skeletal Mascle Mass													
No covariables	11	1.11	0.519	1.709	<0.001					0 (SE = 0.41)		0%	0.920
Participants Characteristics													
Age (years)	11	1.5	−7.901	10.901	0.755	−0.017	−0.424	0.391	0.936	0 (SE = 0.48)	NA%	0%	0.873
Weight (kg)	10	−1.792	−15.819	12.236	0.802	0.038	−0.152	0.227	0.695	0 (SE = 0.56)	NA%	0%	0.863
Height (m)	10	−3.23	−41.849	35.385	0.870	2.37	−19.223	23.964	0.830	0 (SE = 0.55)	NA%	0%	0.853
Training Status	8	2.448	0.418	4.478	0.018	−0.893	−2.259	0.474	0.201	0 (SE = 0.51)	NA%	0%	0.956
Training Characteristics													
Study Durations (weeks)	11	0.885	−0.935	2.704	0.341	0.023	−0.148	0.194	0.794	0 (SE = 0.44)	NA%	0%	0.878
Sessions	11	1.941	−2.455	6.337	0.387	−0.027	−0.171	0.116	0.710	0 (SE = 0.48)	NA%	0%	0.884
Days per week	11	1.824	−0.571	4.219	0.136	−0.217	−0.926	0.492	0.549	0 (SE = 0.44)	NA%	0%	0.900
n exercises per workout	11	3.197	0.25	6.143	0.034	−0.305	−0.728	0.118	0.157	0 (SE = 0.44)	NA%	0%	0.980
Rest between exercise (min)	11	0.719	−0.657	2.095	0.306	0.141	−0.301	0.583	0.532	0 (SE = 0.44)	NA%	0%	0.902
n set per workout	11	1.489	0.304	2.674	0.014	−0.022	−0.082	0.038	0.474	0 (SE = 0.45)	NA%	0%	0.910
range repetitions	7	3.056	−8.385	14.497	0.601	−0.247	−1.593	1.1	0.720	0 (SE = 0.72)	NA%	0%	0.968
Training duration (min)													
Average Intensity (%1RM)													

## Supplementary Materials

The following are available online at https://www.mdpi.com/1660-4601/17/4/1285/s1, Table S1: Main characteristics of the included studies in the present review.
