# Supplementary material for: A Systematic Review with Meta-Analysis of the Effect of Resistance Training on Whole-Body Muscle Growth in Healthy Adult Males
Source: Int J Environ Res Public Health. 2020 Feb 17;17(4):1285. doi: 10.3390/ijerph17041285 (PMC7068252; doi:10.3390/ijerph17041285)
Supplement: Supplementary file 1 [file ijerph-17-01285-s001.pdf]

Supplemental file 1. Main characteristics of the included studies in the present review.

Measure	Study	Hypertrophy measurement	Intervention	N	Age (y)	Initial Weight (kg)	Height (m)	Training Status	Training Type	Study duration (weeks)	Days / week?	n exercises/ workout	Rest between exercise (average min)	n set/ workout	rep	Training duration (min)	Average Intensity(1RM)
FFM	Abe et al. 2000	DXA	ORT	17	37.7	87.6	1.78	Active but resistance untrained	Fullbody	12	3	6		8_12	1_3		65%
FFM	Ahtiainen et al. 2011	Antropometry	ORT	7	28.0	79.0	1.85	Healthy untrained young adult	Fullbody	21	3	8		24	5_20		63%
FFM	Álvarez, et al. 2012	Antropometry	ORT	5	23.9	70.8	1.77	Players with previous experience in RT	Tradicional RT (Split routine)	18	2_3	6	4	12	5_10		78%
FFM	Álvarez, et al. 2012	Antropometry	ORT	5	24.2	68.1	1.72		Tradicional RT (Split routine)	18	2_3	6	4	12	5_10		78%
FFM	Alvehus et al. 2014	DXA	ORT	17	25.3	78.6		Healthy young men with slight to moderate experience in RT	Tradicional RT (Split routine)	8	2_4		1.5		8_15		
FFM	Arazi et al. 2015	Antropometry	RT+PL	10	22.4	74.8	1.75	no RT experience	Fullbody	4	3	9	2	18	8_10		83%
FFM	Arciero et al. 2001	DXA	ORT	10	20.0	78.9	1.83	Active but resistance untrained	Fullbody	4	3	10		10	3		70%
FFM	Arciero et al. 2001	DXA	ORT	10	20.0	78.8	1.79		Fullbody	4	3	10		10	3		70%
FFM	Bang et al. 2017	BIA	ORT	8	33.1	83.8		≥ 3 years of RT experience	Tradicional RT (Split routine)	8	6		1.25	26	10_15		70%
FFM	Bartolomei et al. 2016	Antropometry	ORT	10	23.7	78.5	1.77	≥ 3 years of RT experience	Tradicional RT (Split routine)	15	4	5.5	2	27.5	3_12		70%

FFM	Bartolomei et al. 2016	Antropometry	ORT	8	26.0	78.9	1.70		Tradicional RT (Split routine)	15	4	5.5	2	27.5	3_12		70%
FFM	Bartolomei et al. 2018	Antropometry	ORT	9	24.9	83.5	1.77	≥ 4 years of RT experience	Fullbody	6	4	6	1.5	30	5_4		89%
FFM	Bartolomei et al. 2018	Antropometry	ORT	11	26.0	85.4	1.77		Fullbody	6	4	6	1.5	30	4_12		78%
FFM	Bhasin et al. 1996	Underwater weighing	ORT	9	26.0	85.5	1.81			10	3			6	4_5		80%
FFM	Buresh et al 2009	Underwater weighing	ORT	6	25.3	84.5		Healthy males	Fullbody	10	1_2	16	1	2_3	10		
FFM	Buresh et al 2009	Underwater weighing	ORT	6	21.5	79.8			Fullbody	10	1_2	16	2.5	2_3	10		
FFM	Chromiak et al. 2004	Underwater weighing	ORT	18	22.2	83.6		Active but resistance untrained	Tradicional RT (Split routine)	10	4	6	1.75	3_10	3_4	60	
FFM	Chromiak et al. 2004	Underwater weighing	RT+PL	15	22.1	79.2			Tradicional RT (Split routine)	10	4	6	1.75	3_10	3_4	60	
FFM	Colquhoun et al. 2017	Ultrasound	ORT	14	23.1	79.7		≥ 3 years of RT experience	Fullbody	9	3	5	3.5		1_8		85%
FFM	Crewther et al. 2013	Antropometry	ORT	12	23.6	92.4	1.80	≥ 2 years of RT experience	Fullbody	6	4	6	2.25	21_24	3_12	60	70%
FFM	Deruisseau et al. 2004	Underwater weighing	ORT	13	23.0	78.8	1.79	≥ 1 year of RT experience	Fullbody	13	3	14		8_10	4	60	75%
FFM	Fu et al. 2017	BOD POD	ORT	9	21.0	76.6	1.80	≥ 3 years of RT experience	Fullbody	6	4	9	2	18	8_12	45	75%
FFM	Fu et al. 2017	BOD POD	ORT	9	28.0	79.4	1.78		Fullbody	6	2	9	2	36	8_12	105	75%
FFM	Gallagher et al. 2000	Antropometry	ORT	14	22.3	77.2	1.79	Untrained	Fullbody	8	3	10	1.75	10	3		80%

FFM	Gallagher et al. 2000	Antropometry	ORT	12	21.0	76.1	1.81		Fullbody	8	3	10	1.75	10	3		80%
FFM	Garthe et al. 2013	DXA	ORT	21	19.1	70.9	1.79	Elite athletes	Tradicional RT (Split routine)	10	4	4	2	12_20	6_12		
FFM	Garthe et al. 2013	DXA	ORT	18	19.6	75.0	1.80		Tradicional RT (Split routine)	10	4	4	2	12_20	6_12		
FFM	Ghahramanloo et al. 2009	anthropometry	RT+PL	9	25.4	66.0	175.80	Healthy males	Fullbody	8	3	4			6_10		
FFM	Hong et al. 2014	BIA	ORT	8	23.0	73.7	1.72	Male college students without experience	Tradicional RT (Split routine)	8	3	8	3	21	8_12	50	80%
FFM	Hong et al. 2014	BIA	ORT	10	22.2	71.4	1.76		Tradicional RT (Split routine)	8	3	8	3	21	8_12	50	80%
FFM	Hu et al. 2009	BIA	ORT	48	32.2	81.5	179.80	Untrained men		10	2_3				10_15		70%
FFM	Huso et al. 2002	Underwater weighing	RT+PL	10	24.0	75.2	171.00	Recreationaly actives men	Fullbody	4	3	7_8	1.5	8_10	3		
FFM	Jang et al. 2017	DXA	RT+PL	8	23.3	76.7	1.74	Active	Fullbody	12	3	8	1	24	10_12	45	80%
FFM	Joy et al. 2014	DXA	ORT	14	21.0	75.7	1.75	≥ 1 year of RT experience, ≥3 days/week for the past 6 months	Tradicional RT (Split routine)	8	3	8	1.375	1	6_12		
FFM	Kim et al. 2014	BIA	RT+PL	9	23.6			Untrained healthy young men		12	6					20-50	70%
FFM	Kon et al. 2014	BIA	ORT	7	28.2	65.8	1.70	≥ 10 years of RT experience 2-3 times x week No RT the last 6 months	Tradicional RT (Split routine)	8	2	2	1.5	5	10	20	70%

FFM	Kramer et al. 2009	DXA	RT+PL	9				Healthy, recreationally active men	Tradicional RT (Split routine)	12	3	10		3_5			
FFM	Kreipke et al. 2015	DXA	RT+PL	14	21.0	75.9	1.79	≥ 1 year of RT experience	Tradicional RT (Split routine)	4	4	6	3.5	48	1_10	60	
FFM	Lemmer et al. 2001	DXA	RT+PL	10	25.0	84.4	1.77	Untrained	Fullbody	24	3	7	2.5	15	1_2		
FFM	Li et al. 2014	Antropometry	RT+PL	13	22.3	76.2	1.77	≥ 1 year of RT experience	Tradicional RT (Split routine)	8	3_4	2	1	3	8_12		
FFM	Lockwood et al 2017	DXA	ORT	15	21.8	78.9	1.78	≥ 3 months of RT experience	Tradicional RT (Split routine)	8	4	8	1	24	5_12		
FFM	Lukaski et al. 1996	DXA	RT+PL	12	21.1	79.9	179.30	Untrained	Fullbody	8	5	4		1_12	9	60	75%
FFM	Lukaski et al. 1996	DXA	RT+PL	12	23.3	79.3	177.30	Untrained	Fullbody	8	5	4		1_12	9	60	75%
FFM	Lukaski et al. 1996	DXA	RT+PL	12	22.3	79.2	178.00	Untrained	Fullbody	8	5	4		1_12	9	60	75%
FFM	Mazzetti et al. 2000	Antropometry	RT+PL	10	25.2	85.9	1.76	≥ 2 years of RT experience	Fullbody	12	3_4	7_8	2	3_12	2_4		
FFM	Mazzetti et al. 2000	Antropometry	RT+PL	8	23.8	84.5	1.78	≥ 2 years of RT experience	Fullbody	12	3_4	7_8	2	3_12	2_4		
FFM	McCarthy et al. 1995	Antropometry	RT+PL	10	27.9	82.0		Untrained	Fullbody	10	3	8	1.25	6	3	50	
FFM	Nacleiro et al. 2017	BOD POD	RT+PL	8	25.0	76.9	1.77	≥ 2 years of RT experience	Circuit	8	3	8	1.5	24	12	30	
FFM	Noonan et al. 1998	Underwater weighing	RT+PL	13	19.4	105.9	1.86	NCAA Football team	Tradicional RT (Split routine)	8	4	7	2.25	2_10	3	60	
FFM	Oliver et al. 2013	DXA	ORT	11	25.0	81.7	1.80	RT ≥ once a week the last 2 years	Fullbody	12	4	8	1.75	32	10		68%
FFM	Oliver et al. 2013	DXA	ORT	11	25.0	82.5	1.80		Fullbody	12	4	8	1.25	32	5_10		68%
FFM	Ormsbee et al. 2012	DXA	RT+PL	11	24.0	83.7		RT trained (≥2× week; training for	Tradicional RT	6	3	8	1.25	24	4_10		80%

								≥12 months). 5,3 years	(Split routine)								
FFM	Ormsbee et al. 2013	DXA	RT+PL	11	23.6	82.2	1.81	RT trained (≥3× per week; training for ≥12 months). 4,5 years	Tradicional RT (Split routine)	6	3	8	1.5	24	4_10		80%
FFM	Pérez-Gómez et al. 2013	DXA	RT+PL	8	22.0	70.4	1.75	Healthy and physically active		10		5	1.5				70%
FFM	Piirainen et al. 2008	BIA	ORT	6	19.1	70.7	1.82	Healthy Finnish military male conscripts	physical military training	8							
FFM	Piirainen et al. 2008	BIA	ORT	6	18.8	72.8	1.82		physical military training	8							
FFM	Radaelli et al. 2015	Antropometry	ORT	12	24.1	79.7	1.77	Active but resistance untrained	Fullbody	24	3	10	1.75	10	8_12	60	
FFM	Radaelli et al. 2015	Antropometry	ORT	13	24.1	76.2	1.74	Active but resistance untrained	Fullbody	24	3	10	1.75	30	8_12	60	
FFM	Radaelli et al. 2015	Antropometry	ORT	13	24.7	82.2	1.72		Fullbody	24	3	10	1.75	50	8_12	60	
FFM	Ribeiro et al. 2015	DXA	ORT	5	26.6	84.6	no	Professional bodybuilding competitors	Fullbody	4	4	10.5	1.5	42	6_12	95	
FFM	Ribeiro et al. 2015	DXA	ORT	5	26.8	85.9	no		Tradicional RT (Split routine)	4	6	8	1.5	32	6_12	65	
FFM	Roberts et al. 2007	DXA	ORT	16		87.1		Healthy male resistance	-	-	-	-				
FFM	Rogerson et al. 2007	BIA	RT+PL	11	19.0	87.6	180.70	Elite male rugby league players	Fullbody	20 sessions	5	5-6/per day		2	2_8 and day for whole body power 1_4		
FFM	Schumann et al. 2014	DXA	ORT	16	30.0	78.0	1.79	Moderately physically active	Tradicional RT (Split routine)	24	2_3	9	2	2_5	8_10	30-50	83%

FFM	Schumann et al. 2014	DXA	ORT	18	30.0	78.0	1.79		Tradicional RT (Split routine)	24	2_3	9	2	2_5	8_10	30-50	83%
FFM	Shelmadine et al. 2009	DXA	RT+PL	9	22.8	79.3	179.49	Healthy, recreationally active without RT experience	Tradicional RT (Split routine)	7	4	16		2 sets / 2 exercise	10		
FFM	Snijders et al. 2015	DXA	ORT	19	21.0	80.0	1.85	Active	Fullbody	12	3	4	2.25	12	8_15		75%
FFM	Spence et al. 2011	DXA	ORT	13	26.6	81.7	1.81	Healthy, untrained male	Tradicional RT (Split routine)	24	3		1.5	2_3	12_15		75%
FFM	Spillane et al. 2009	DXA	RT+PL	10	20.2	77.9	175.39	Healthy males (at least twice weekly RT for 1 year prior to the study	Tradicional RT (Split routine)	7	4	16	2	3	8_10		75%
FFM	Spillane et al. 2011	DXA	RT+PL	19	22.8	79.1	1.79		Tradicional RT (Split routine)	4	4	8	2	24	10		75%
FFM	Terzis et al. 2008	DXA	ORT	8	21.0	83.0	181.00		Fullbody	14	2_3	9		2_3	6_10		
FFM	Terzis et al. 2008b	DXA	ORT	11	22.0	85.0	1.84	Male physical education students	Fullbody	14	2_3	9		2_3 sets; 3_4 minutes	8RM_ 10RM; 6RM;		
FFM	Thomson et al. 2009	BIA	ORT	17	24.0	85.0	1.80	≥ 1 year of RT experience	Fullbody	9	3	9		3 sets	5_15		
FFM	Tomljanović et al. 2011	BIA	RT+PL	23	22-25	82.4	1.85	≥ 6 months of RT experience	Fullbody	5	3	4		16	6_10		80%
FFM	Wilborn, et al. 2010	Underwater weighing	RT+PL	13	21.0	84.0	1.80	Resistance-trained	Tradicional RT (Split routine)	8	4	16		3/exercise and 2/sets	5_10		
FFM	Willoughby et al. 2007	Underwater weighing	RT+PL	10	19.0	81.5	1.75	Untrained	Fullbody	10	4	10		3	6_8	45	

FFM	Willoughby et al. 2014	DXA	RT+PL	10	21.4	84.3	1.81	Training at least twice weekly. RT for one year prior to the study.	Tradicional RT (Split routine)	4	4	9	2	27	10		75%
FFM	Wilson et al. 2013	DXA	RT+PL	10	23.7	85.7	1.79	Resistance-trained males	Tradicional RT (Split routine)	12	3	10		30	3_14		
FFM	Wilson et al. 2014	DXA	ORT	9	21.6	87.1	1.81		Tradicional RT (Split routine)	12	3_5	11		3_5	3_14		
FFM	Zamani et al. 2017	BIA	ORT	10	21.2	66.5	1.76	Untrained	Fullbody	8	3	6	2	24	6_8		83%
FFM	Zamani et al. 2017	BIA	ORT	10	24.2	70.0	1.70	Untrained	Tradicional RT (Split routine)	8	3	6	2	24	6_8		83%
FFM	Zamani et al. 2017	BIA	ORT	10	21.0	65.1	1.74	Untrained	Tradicional RT (Split routine)	8	3	6	2	24	6_8		83%
LMM	Alcaraz et al. 2011	DXA	ORT	11	22.7	75.2	1.76	≥ 1 year of RT experience	Fullbody	8	3	6	3	27	6	105_125	88%
LMM	Alcaraz et al. 2011	DXA	ORT	15	22.7	75.2	1.76		Circuit	8	3	6	0	27	6	55_78	88%
LMM	Alvehus et al. 2014	DXA	ORT	17	25.3	76.8		Healthy young men with slight to moderate experience in RT	Tradicional RT (Split routine)	8	2_4		1.5		8_15		
LMM	Andre et al. 2016	DXA	ORT	9	20.2	84.6	1.79	≥ 3 years of RT experience	Tradicional RT (Split routine)	8	4	8	2	24	4_12		75%
LMM	Ara et al. 2007	DXA	ORT	12	22.7	74.7	1.74	Familiar with strength training	lower limbs	6	3	5			3_10		70%
LMM	Bemben et al. 2001	Underwater weighing	ORT	8	19.0	95.7	1.88	NCAA Soccer players	Fullbody	9	4	10	4	8_12/2_4		75	85%

LMM	Brown et al. 1999	Underwater weighing	ORT	10	23.3	81.1	1.78	Untrained	Fullbody	8	3	10	3	8_10		83%
LMM	Burke et al. 2001	DXA	RT+PL	5	18_31	80.0		4.2 years of RT experience	Tradicional RT (Split routine)	6	4	7_9	1.5	6_12		
LMM	Caldow et al. 2015	DXA	ORT	10	21.2	75.4	1.80	≥ 6 months of RT experience	Fullbody	12	3	8	1	24	8_12	80%
LMM	Chycki et al. 2016	DXA	ORT	6	21.0	80.6	1.79		Fullbody	6	2	2	3	16	10	70%
LMM	Chycki et al. 2016	DXA	ORT	6	22.0	81.1	1.77	≥ 3 years of RT experience	Fullbody	6	2	2	3	16	10	70%
LMM	Chycki et al. 2016	DXA	RT+PL	6	22.0	81.1	1.77		Fullbody	6	2	2	3	16	10	70%
LMM	Deyssig et al. 1993	Anthopometry	ORT	11	23.4			≥6 months of RT experience		6						
LMM	Escalante et al. 2016	DXA	ORT	10	25.6	88.6	1.74	≥1 year of RT experience	Fullbody	8	3	6	3		1_12	
LMM	Fahey and Brown 1973	Underwater weighing	RT+PL	13		75.0		≥1 year of RT experience	Fullbody	9	3	5	4	5		
LMM	Falk et al. 2003	Underwater weighing	RT+PL	15	22.7	88.3	179.80	≥2 years of experience	Tradicional RT (Split routine)	8	4	5_14		5_12		70%
LMM	Falk et al. 2003	Underwater weighing	RT+PL	13	21.8	82.9	179.40		Tradicional RT (Split routine)	8	4	5_14		5_12		70%
LMM	Fyfe et al. 2016	DXA	ORT	8	29.5	82.6	1.81	Active	Fullbody	8	3	5	2.5	20	4_14	78%
LMM	Garthe et al. 2013	DXA	ORT	21	19.1	70.9	1.79	Elite athletes (3.8 h x week strength training)	Tradicional RT (Split routine)	10	4	4	2	12_20	6_12	
LMM	Garthe et al. 2013	DXA	RT+PL	18	19.6	75.0	1.80		Tradicional RT (Split routine)	10	4	4	2	12_20	6_12	

LMM	Glowacki et al. 2004	Underwater weighing	ORT	13	23.0	72.8	1.76	Untrained	Fullbody	12	2_3	8	3	6_10			80%
LMM	Gobbo et al. 2013	DXA	ORT	15	23.1	72.2	1.78	University students, engaged in RT programs during the last 4 months before the study.		8	4	8	1.5	32	6_12		
LMM	Harber et al. 2004	Underwater weighing	ORT	8	23.6	80.9		Untrained	Circuit	10	3	10					50%
LMM	Hoffman et al. 2009	DXA	ORT	13	19.6	102.3	1.83	Strength and power athlete	Tradicional RT (Split routine)	10	4	20		3_4	4_10		
LMM	Hoffman et al. 2012	DXA	ORT	9	22.5	89.4	1.80	≥1 year of training experience	Tradicional RT (Split routine)	8	4	9_10	1.5	29	10_12		70%
LMM	Ihalainen et al. 2017	DXA	ORT	31		81.1		Active but RT untrained	Fullbody	12	2.5	9	2	22.5	4_12		73%
LMM	Ihalainen et al. 2017	DXA	RT+PL	37		84.9			Fullbody	12	2.5	9	2	22.5	4_12		85%
LMM	Joy et al. 2016	DXA	RT+PL	11				Resistance-trained	Fullbody	10	3	8	2.75	32	1_12	60	80%
LMM	Joy et al. 2016	DXA	RT+S	10	27.2	82.8	1.73		Fullbody	10	3	8	2.75	32	1_12	60	80%
LMM	Kelly et al, 1998	Underwater weighing	ORT	9	26.8			≥2 years of RT experience	Tradicional RT (Split routine)	4	2	4	3	6_10			
LMM	Kerksick et al 2009	DXA	ORT	24	19.8	81.3	1.79	Healthy male	Tradicional RT (Split routine)	8	4	14_16	1	3	8_10		
LMM	Kerksick et al. 2006	DXA	ORT	11		85.1		Healthy male	Fullbody	10	4	7_8	1.5	3	6_10		80%
LMM	King et al. 1999	Underwater	RT+PL	9	19_29	80.6	176.80	Not currently engaged in RT	Fullbody	8	3	10	3	8_10			83%

		weighing															
LMM	Ko and Choi 2013	BIA	ORT	18	23.8	76.9	1.75	University students		8	3	9		36	8_15	30_60	65%
LMM	Kreider et al. 2002	DXA	ORT	23	23.0	82.0	1.79	≥1 year of RT		4			3	6_10			
LMM	Lemon et al. 1992	Underwater weighing	ORT	12	22.4	81.9	1.81	Active but without RT experience	Tradicional RT (Split routine)	4	6	5_8	4	10			78%
LMM	lo et al. 2011	DXA	ORT	10	20.2	65.0	1.70	Nonathletic male students	Fullbody	24	3	11		16.5	4_15		83%
LMM	Lowery et al. 2014	DXA	RT+PL	12	21.3			≥ 1 year RT	Tradicional RT (Split routine)	8	3	7_8	1.5	21_24	6_15		
LMM	Mangine et al. 2008	anthropometry	ORT	8	21.4	82.7	176.80	Previous lifting experience	Fullbody	8	3	12_16	2	3_5	3_10		55%
LMM	Mangine et al. 2008	anthropometry	ORT	9	20.1	81.0	180.80		Fullbody	8	3	12_16	2.5	3_4	6_10		85%
LMM	Mayhew et al. 2009	DXA	ORT	21	27.9	75.4	170.40	Healthy untrained	lower limbs	16	3	3?		3	8_12		
LMM	Miller et al. 1984	Underwater weighing	RT+PL	8		70.5		Untrained students	Fullbody	10	3	10	3	8		90_120	
LMM	Moore et al. 2007	DXA	RT+PL	12	22.0	78.5		recreatonally active (not engaged in any RT)	Fullbody	12	5	13		2_3	6_12		83%
LMM	Nybo et al. 2010	DXA	ORT	8	36.0	95.0		Untrained men	lowr limbs	12	2_3	6			6_16	60	
LMM	Oliver et al. 2013	DXA	ORT	11	25.0	81.7	1.80	RT at least once a week for the previous 2 years. 6.5 years trained	Fullbody	12	4	8	1.75	32	10		68%
LMM	Oliver et al. 2013	DXA	RT+PL	11	25.0	82.5	1.80	RT at least once a week for the previous 2 years. 6.0 years trained	Fullbody	12	4	8	1.25	32	5_10		68%
LMM	Paoli et al. 2017	DXA	ORT	18	23.5	80.0	1.82		Fullbody	8	3	4.5	1.75	18	12_18		

LMM	Paoli et al. 2017	DXA	ORT	18	25.5	80.7	1.85	Soccer players without experience	Fullbody	8	3	4	2.75	16	6_8		
LMM	Peeters et al. 1999	Anthopometry	ORT	14	21.2	86.4	1.78	≥2 years of RT experience	Tradicional RT (Split routine)	6	4	3	5	4_10			78%
LMM	Pérez-Gómez et al. 2013	DXA	ORT	8	22.0	70.4	1.75	Healthy and physically active		10		5	1.5				70%
LMM	Peronnet et al. 1986	Underwater weighing	RT+PL	7	19.0	80.0	1.76	Actives	Fullbody	8	3	7	3	5	2	45	
LMM	Saremi et al. 2010	DXA	RT+PL	8	23.9	77.6	1.80	Healthy non-RT young men	Fullbody	8	3	6	2	3	8_10		65%
LMM	Schneider et al. 2003	DXA	ORT	7	33.0	78.0	180.00	Untrained	Fullbody	16	3	3	4	10			60%
LMM	Schumann et al. 2014	DXA	ORT	16	30.0	78.0	1.79	Moderately physically active	Tradicional RT (Split routine)	24	2_3	9	2	2_5	8_10	30-50	83%
LMM	Schumann et al. 2014	DXA	ORT	18	30.0	78.0	1.79		Tradicional RT (Split routine)	24	2_3	9	2	2_5	8_10	30-50	83%
LMM	Slater et al. 2001	DXA	ORT	7	24.9	89.7	1.92	7.4 years of experience	Not specified	6	2_3		4	4_6			
LMM	Spence et al. 2011	DXA	ORT	13	26.6	81.7	1.81	Untrained	Tradicional RT (Split routine)	24	3		1.5	2_3	12_15		75%
LMM	Spillane et al. 2016	DXA	ORT	11	19.4	86.1	1.79	≥ 1 year	Tradicional RT (Split routine)	8	4	8	2	24	8_10		75%
LMM	Taylor et al. 2011	DXA	RT+PL	17	21.0	85.0	1.79	RT trained	Tradicional RT (Split routine)	8	4		2.5		8_10		75%
LMM	Thorstensson et al. 1976	DXA	RT+PL	14	19-31	74.2		Active but RT untrained		8	3		3	6			

LMM	Tinsley et al. 2017	DXA	ORT	18	22.0	79.0	1.80		Tradicional RT (Split routine)	8	3	5.5	1.5	22	8_12	
LMM	Volek et al. 2004	DXA	RT+PL	9	20.7	86.7	1.79	5.1 (3) years of experience	Fullbody	4	5	8	4	3_12		
LMM	Yan et al. 2016	DXA	ORT	8	22.2	70.5	1.76		Fullbody	5	2	1	1	5	10	70%
LMM	Yan et al. 2016	DXA	ORT	9	22.2	70.5	1.76	Active but RT untrained		5	2	1	1	5	10	70%
LMM	Yan et al. 2016	DXA	ORT	8	22.2	70.5	1.76			5	2	1	1	5	10	70%
SMM	Álvarez, et al. 2012	Antropometry	ORT	5	23.9	70.8	1.77	Players with previous experience in RT	Tradicional RT (Split routine)	18	2_3	6	4	12	5_10	78%
SMM	Álvarez, et al. 2012	Antropometry	ORT	5	24.2	68.1	1.72		Tradicional RT (Split routine)	18	2_3	6	4	12	5_10	78%
SMM	Gobbo et al. 2013	Antropometry	ORT	15	23.1	72.2	1.78		Tradicional RT (Split routine)	8	4	8	1.5	32	6_12	
SMM	Gobbo et al. 2013	Antropometry	ORT	15	23.1	72.2	1.78	University students, engaged in RT programs during the last 4 months before the study.	Tradicional RT (Split routine)	8	4	8	1.5	32	6_12	
SMM	Gobbo et al. 2013	Antropometry	ORT	15	23.1	72.2	1.78		Tradicional RT (Split routine)	8	4	8	1.5	32	6_12	
SMM	Gobbo et al. 2013	Antropometry	RT+PL	15	23.1	72.2	1.78		Tradicional RT (Split routine)	8	4	8	1.5	32	6_12	
SMM	Lockwood et al 2017	DXA	RT+PL	15	21.8	78.9	1.78	≥ 3 months of RT experience	Tradicional RT	8	4	8	1	24	5_12	

									(Split routine)							
SMM	Maksimović et al 2016	Antropometry	ORT	15	22	78.92	1.81	Recreationally active	Fullbody	12	3	6	4	6_12		88%
SMM	Mobley et al 2017	DXA	ORT	15	21	81	1.82	Recreationally active	Fullbody	12	3	4	5	4_10		
SMM	Nunes et al 2017	DXA	RT+PL	22	22.2	73.1	1.78	≥ 1.5 years of RT experience	Fullbody	8	4	9	4	6_12	1_2	
SMM	Paoli et al 2015	Antropometry	RT+PL	19	24.9				Fullbody	8	3	6	3	6_12	3	

FFM: Fat Free Mass; SMM: Skeletal muscle mass: LMM: Lean muscle mass; DXA: Dual X ray Absorptiometry; BIA: Bioelectrical impedance analysis; RT: resistance training; PL: Placebo; ORT: Only Resistance Training; S: Supplementation; 1-RM: One repetition maximum.
